# Prediction of complications and surgery duration in primary TKA with high accuracy using machine learning with arthroplasty-specific data

**DOI:** 10.1007/s00167-022-06957-w

**Published:** 2022-04-08

**Authors:** Florian Hinterwimmer, Igor Lazic, Severin Langer, Christian Suren, Fiona Charitou, Michael T. Hirschmann, Georg Matziolis, Fritz Seidl, Florian Pohlig, Daniel Rueckert, Rainer Burgkart, Rüdiger von Eisenhart-Rothe

**Affiliations:** 1 Department of Orthopaedics and Sports Orthopaedics, Klinikum Rechts der Isar, Technical University of Munich, Munich, Germany; 2 Institute for AI and Informatics in Medicine, Technical University of Munich, Munich, Germany; 3 Department of Trauma Surgery, Klinikum Rechts der Isar, Technical University of Munich, Munich, Germany; 4 Department of Orthopaedic Surgery and Traumatology—Liestal, Kantonsspital Baselland, Bruderholz, Laufen, Switzerland; 5 Orthopaedic Department Campus Eisenberg, University Hospital Jena, Eisenberg, Germany; 6 Endoprosthetics Committee of the German Knee Society (DKG), Munich, Germany

**Keywords:** Artificial intelligence, Machine learning, Knee surgery, Total knee arthroplasty, Knee arthroscopy, Supervised learning

## Abstract

**Purpose:**

The number of primary total knee arthroplasties (TKA) is expected to rise constantly. For patients and healthcare providers, the early identification of risk factors therefore becomes increasingly fundamental in the context of precision medicine. Others have already investigated the detection of risk factors by conducting literature reviews and applying conventional statistical methods. Since the prediction of events has been moderately accurate, a more comprehensive approach is needed. Machine learning (ML) algorithms have had ample success in many disciplines. However, these methods have not yet had a significant impact in orthopaedic research. The selection of a data source as well as the inclusion of relevant parameters is of utmost importance in this context. In this study, a standardized approach for ML in TKA to predict complications during surgery and an irregular surgery duration using data from two German arthroplasty-specific registries was evaluated.

**Methods:**

The dataset is based on two initiatives of the German Society for Orthopaedics and Orthopaedic Surgery. A problem statement and initial parameters were defined. After screening, cleaning and preparation of these datasets, 864 cases of primary TKA (2016–2019) were gathered. The XGBoost algorithm was chosen and applied with a hyperparameter search, a cross validation and a loss weighting to cope with class imbalance. For final evaluation, several metrics (accuracy, sensitivity, specificity, AUC) were calculated.

**Results:**

An accuracy of 92.0%, sensitivity of 34.8%, specificity of 95.8%, and AUC of 78.0% were achieved for predicting complications in primary TKA and 93.4%, 74.0%, 96.3%, and 91.6% for predicting irregular surgery duration, respectively. While traditional statistics (correlation coefficient) could not find any relevant correlation between any two parameters, the feature importance revealed several non-linear outcomes.

**Conclusion:**

In this study, a feasible ML model to predict outcomes of primary TKA with very promising results was built. Complex correlations between parameters were detected, which could not be recognized by conventional statistical analysis. Arthroplasty-specific data were identified as relevant by the ML model and should be included in future clinical applications. Furthermore, an interdisciplinary interpretation as well as evaluation of the results by a data scientist and an orthopaedic surgeon are of paramount importance.

**Level of evidence:**

Level IV.

## Introduction

With the growing functional demands of patients and increasing advances in arthroplasty, the number of primary total knee arthroplasties (TKA) is expected to rise continuously [20, 27]. Although the percentage of early revisions in TKA is only between 2 and 3%, the absolute number of patients who have to undergo revision will increase dramatically [5, 23, 26]. Revision TKA is a more demanding procedure and is associated with higher costs and often inferior outcomes than primary TKA [9]. Hence, the early identification of patients at risk for revision will become increasingly relevant. Risk adjustment tools with clinical applicability will therefore gain importance as the rising incidence of primary and revision procedures will require patients and health care providers to make decisions when faced with the individual odds of unfavourable outcomes and the necessity of resource allocation.

Considerable efforts have already been made towards the development of risk stratification models based on administrative and medical registry data. However, the prediction of adverse events has so far been moderately accurate and the external validation is difficult to achieve [10, 22]. Risk adjustment as a part of precision medicine will play an increasingly important role in the future of arthroplasty and novel, more comprehensive and specific risk analysis tools are urgently needed. Machine learning (ML) represents a distinct application of artificial intelligence (AI), which evolved from pattern recognition and learning theory. ML is just in its early stages in orthopaedics and standardized approaches are not yet established. ML was recently applied and evaluated in predicting adverse events in TKA with heterogeneous results: El Galaly et al. built models to predict the likelihood of revision TKA within 2 years using data from the Danish Knee Arthroplasty Registry [11]. Similarly, Harris et al. applied machine learning methods using large administrative databases to develop and validate prediction models for mortality and complications after total joint arthroplasty, yielding moderate results. Both studies highlighted that no clinically useful prediction model was achievable. The inherent difficulty in predicting rare outcomes—such as adverse events in TKA—is based on the limitation of the applied data. ML may be capable to expose unseen patterns in large datasets. However, from a data science perspective, the data volumes of tabular data in arthroplasty are relatively small. Hence, the data sources must be chosen carefully so that the presence of relevant patterns in the dataset is warranted in the first place. The selection and inclusion of relevant input parameters is therefore of utmost importance to allow patterns that are specific to TKA. The use of arthroplasty-specific databases in this context appears to be crucial.

Furthermore, pattern recognition cannot be immediately transformed into constellations of specific risk factors. These patterns must subsequently be interpreted in the medical context, especially when occurrence of the predicted outcome is rare, as already demonstrated by El Galaly et al. [11]. In this context, consideration must be given to the extent to which a specific outcome such as revision can be replaced with a simpler surrogate. It was hypothesized by us that the matching of the outcome with specific input parameters and the corresponding adjustment of the ML algorithm is critical to improve the predictive accuracy of ML algorithms in TKA. Since all of these considerations require an extensive knowledge about statistics, information technology and orthopaedics, an interdisciplinary collaboration between data scientists and orthopaedic surgeons is essential to the success of the ML model. The development of clinically applicable risk analysis models is of high clinical relevance and ML is perfectly suited for this task. In this study, a standardized approach for ML in TKA with inclusion of data from two German arthroplasty-specific registries was evaluated to investigate if ML with arthroplasty-specific data is feasible and if arthroplasty-specific data increases the performance of such algorithms.

## Material and methods

This study was approved by an institutional ethics commission under no. 714/20 S (Klinikum rechts der Isar, Technical University of Munich).

Following a standardized approach for ML model development, initially relevant data sources were ascertained and significant parameters to the research question by a literature review were identified. After a thorough data cleaning and preparation, the data was screened to assess potential objectives of the ML model. Subsequently, these objectives were specified and defined the corresponding outcome labels. An ML algorithm was chosen and developed accordingly. For outcome evaluation, several metrics were defined a priori. To increase the quality of the presented observational study and its prediction model, it was reported in accordance with the Transparent Reporting of a Multivariable Prediction Model for Individual Prognosis or Diagnosis (TRIPOD) guidelines [8] and the Strengthening the Reporting of Observational Studies in Epidemiology (STROBE) statement [28].

### Data source

The German Society for Orthopaedics and Orthopaedic Surgery (“Deutsche Gesellschaft für Orthopädie und Orthopädische Chirurgie” (DGOOC)) has introduced two initiatives to improve the quality of care. The German Arthroplasty Registry (Endoprothesenregister Deutschland (EPRD)) reports procedure and implant-related data of hip and knee replacements. EndoCert is a certification process of medical facilities in the field of joint replacement and is used to monitor compliance with structural, process and outcome quality standards in hospitals. To ensure the reproducibility of this study in other clinics, the results of this study are based on the retrospective datasets provided to these two initiatives by our institution. The data presented were collected exclusively at Klinikum rechts der Isar (Munich) and include all primary TKA cases performed at our institution from 2016 to 2019.

### Parameters and data screening

The parameters from EndoCert and EPRD were screened collaboratively a priori by a data scientist (F.H.) and an orthopaedic surgeon (I.L.) regarding their relevance and applicability for ML analysis. The complete dataset consists of 864 patient cases from our hospital. Discrete parameters are presented by their distribution and continuous variables by their mean, standard deviation, and variance. Tables 1 and 2 illustrate all discrete and continuous input parameters and their sources. It comprises data from the year 2016 (25.6%), 2017 (35.8%), 2018 (17.1%) and 2019 (21.5%).

**Table 1:** Data description: discrete parameters

Patients	864	100%	Data source
Parameters	Absolute	Relative	
Year			EndoCert / EPRD
2016	221	25.6%	
2017	309	35.8%	
2018	148	17.1%	
2019	186	21.5%	
Sex			EndoCert / EPRD
Male	376	43.5%	
Female	488	56.5%	
Diagnosis			EndoCert / EPRD
Primary osteoarthritis	769	89.0%	
Post-traumatic osteoarthritis	46	5.3%	
Retropatellar osteoarthritis	14	1.6%	
Aseptic osteonecrosis	5	0.6%	
Tumour/metastasis	29	3.4%	
Fracture	1	0.1%	
Side			EndoCert / EPRD
Left	422	48.8%	
Right	442	51.2%	
Implant type			EPRD
Primary implant	743	86.0%	
Revision implant	94	10.9%	
Tumour implant	27	3.1%	
Surgeon			EndoCert
Surgeon 1	240	27.8%	
Surgeon 2	110	12.7%	
Surgeon 3	229	26.5%	
Surgeon 4	72	8.3%	
Surgeon 5	85	9.8%	
Surgeon 6	44	5.1%	
Surgeon 7	43	5.0%	
Surgeon 8	3	0.3%	
Surgeon 9	19	2.2%	
Surgeon 10	15	1.7%	
Surgeon 11	2	0.2%	
Surgeon 12	2	0.2%	
Experience level of surgeon			EndoCert
1 Resident	1	0.1%	
2 Fellow	48	5.6%	
3 Attending—junior	0	0.0%	
4 Attending—main surgeon*	156	18.1%	
5 Attending—senior surgeon*	659	76.3%	
Surgery type			EndoCert / EPRD
1 Primary arthroplasty	720	83.3%	
2 Mobile component exchange	10	1.2%	
3 Revision arthroplasty	124	14.4%	
4 REVISION SURGERY	10	1.2%	

^*^ “Attending—main surgeon” corresponds to “Hauptoperateur” and “Attending—senior surgeon” corresponds to “Senior-Hauptoperateur” as defined by Endocert

**Table 2:** Data description of continuous parameters

Patients	864	100%		
Parameters	Mean	Std. deviation	Variance	Data scource
Age	66.5	11.9	141.4	EndoCert / EPRD
Height (in cm)	170.4	11.1	123.6	EPRD
Weight (in kg)	84.5	20.5	421.7	EPRD
BMI	29.8	16.6	274.7	EPRD

### Data cleaning and preparation

In total, 5.7% (693/12096) of data points were missing and could not be retrieved from the clinical information system. 26.7% (231/864) of data points regarding weight, height and therefore BMI are missing, because they were added to the EPRD registry only since 2017. The data samples were still kept for the final dataset and a model, which can cope with limited missing data points, was chosen.

### Specification of the output labels

The presented model aimed to identify “difficult to treat” cases for primary TKA. After thorough screening of the data, the occurrence of any complication was defined in a binary classification as the primary outcome label. Complications occurred in 6.3% (54/864) cases. Therefore, the definition of complications according to EndoCert (within 90 days after implantation) was used:

- Deviation mechanical axis ± 3°
- Periprosthetic infection
- Dislocation
- Periprosthetic fracture
- Revision surgery
- Thromboembolism
- Neurologic complications
- Mortality

To translate the optimization problem into a binary classification task, the cases were categorised as “no complication occurred” or “at least one complication occurred”. Additionally, as a further surrogate for complex cases, it was aimed to predict irregular durations of surgery in a binary classification which occurred in 11.5% (99/864). According to EndoCert, the duration of a TKA surgery is irregular, if the duration is either < 40 min or > 120 min.

### Hardware and software

Model training and inference was conducted on a common Fujitsu Celsius W580power with 16 GB RAM (Minato City, Tokyo, Japan) and an Intel(R) Xeon(R) E-2124 CPU (3.30 GHz). Implementation of the code was realized with Python 3.9.6 (https://www.python.org) and the Scikit-learn library (https://scikit-learn.org). The source code for this study is provided on GitHub (https://github.com/FlorianH3000/ML_tabdata).

#### Algorithm

**Fig. 1:**
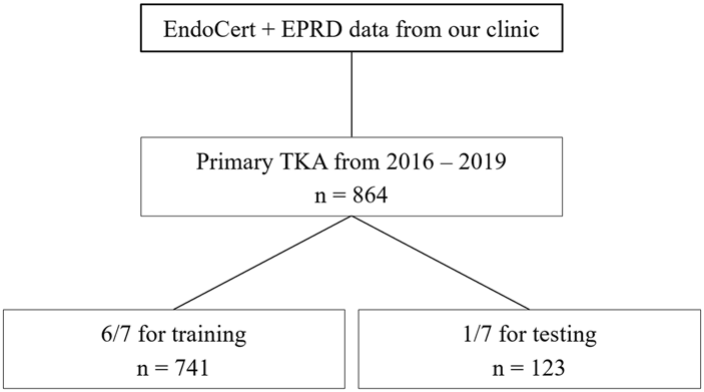
Flowchart describing training and testing datasets

**Fig. 2:**
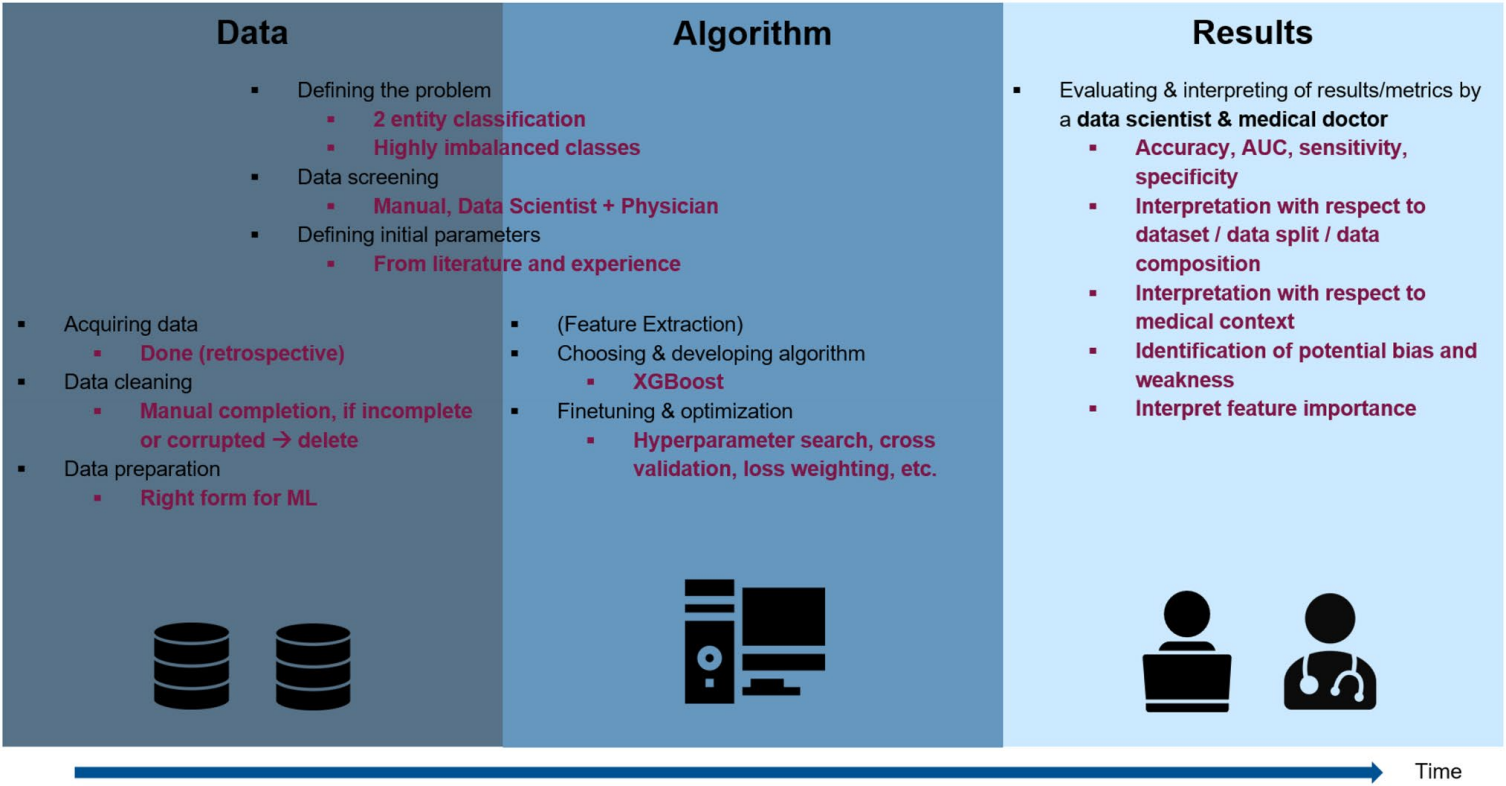
Overview of algorithm development (adapted from [10])

The XGBoost algorithm was chosen [3, 7]. XGBoost is a modern implementation of gradient boosting decision trees and designed for speed, performance and managing missing data points. To provide statistical significance, a cross-validation was applied, where the data was split into a specific number of folds. At least onefold is used solely for testing, and the other folds for training. All folds must be disjunct to avoid cross contamination. After several runs, each data sample was used for testing exactly once. Then, the results were averaged to obtain more realistic and stable metric values. For this study, a hyperparameter search for the optimal number of folds (data split) was performed resulting in a split of seven folds (1 for testing, 6 for training) (Fig. 1). To tackle the significant class imbalance, a loss weighting was applied. The loss of the entity with less samples (in this study the occurrence of complications / irregular duration of surgery) was weighted higher than the dominant entities (no complications / normal duration of surgery). A feature importance was calculated to support deeper understanding of the algorithm’s predictions and give insight into the data. Feature importance refers to the technique that assigns a score to input parameters based on how useful they are at predicting a target. Figure 2 displays the overall approach and fine-tuning techniques.

### Statistical analysis

**Fig. 3:**
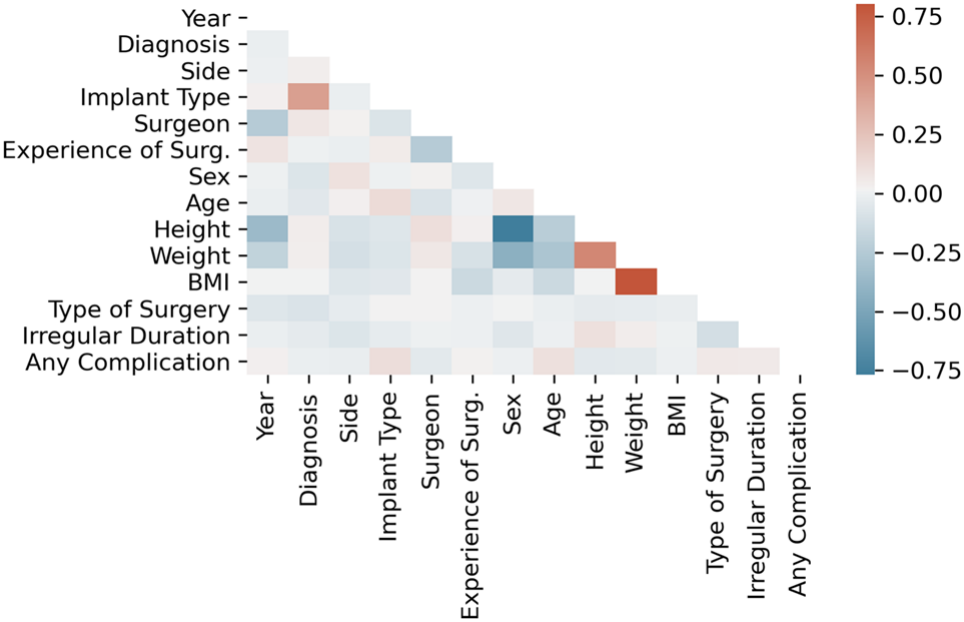
Correlation matrix of all parameters

None of the parameters were normally distributed according to normality test by D’Agostino–Pearson. Figure 3 shows a correlation matrix according to values of Spearman’s rank-order correlation coefficient, which is a measure for linear correlation between two datasets and does not assume that both datasets are normally distributed. A |ρ|> 0.5 concludes a significant direct or indirect correlation between two parameters. None of the relations fulfils such a ρ value (indicated by the scale on the right), except for ‘weight’/ ‘BMI’ (ρ = 0.80), ‘height’/ ‘sex’ (ρ = − 0.77) and ‘weight’/ ‘height’ (ρ = 0.55).

## Results

Eight-hundred and sixty-four patient cases and the respective events from 2016 to 2019 were evaluated in this retrospective dataset. The mean patient age is 66.5 ± 11.9 years. 48.8% of interventions were conducted on the patients’ left side, 51.2% on the right side. The BMI resulted in a mean of 29.8 ± 16.6.

Indications for TKA were classified as primary osteoarthritis (89.0%), posttraumatic osteoarthritis (5.3%), tumour/metastasis (3.4%), retropatellar osteoarthritis (1.6%), aseptic osteonecrosis (0.6%) and fracture (0.1%). The surgeries were performed by 12 different surgeons with a share of 0.2% up to 27.8% of all 864 surgeries. The experience level of surgeons specified by the EndoCert initiative was distributed from 0.1% (level 1), 5.6% (level 2), 0% (level 3), 18.1% (level 4) to 76.3% (level 5). The following surgeries were performed: primary arthroplasties (83.4%), mobile component exchange (1.2%), revision arthroplasties with component exchange (14.4%) and subsequent revision surgery without component exchange (1.2%).

Table 3 displays the distribution of the outcome labels. At least one complication occurred in 6.3% (54/864) of all patients. An irregular duration of surgery (< 40 min or > 120 min) resulted in 11.5% (99/864) of cases.

**Table 3:** Outcome labels

Patients	864	100%	
Parameters	Absolute	Relative	Data source
Complications	(any)		EndoCert
Yes	54	6.3%	
No	810	93.8%	
Irregular duration of surgery	(< 40 min or > 120 min)		EndoCert
Yes	99	11.5%	
No	765	88.5%	

Sixty-three complications in 54 cases occurred (6 cases with multiple complications) in total: 20 (2.3%) deviations from the mechanical axis, 10 (1.2%) periprosthetic infections, 0 (0.0%) dislocations, 4 (0.5%) periprosthetic fractures, 17 (2.0%) revisions, 7 (0.8%) thromboembolism, 2 (0.2%) neurologic complications and 3 (0.3%) deaths. Surgery duration ranged from 11 to 386 min.

### Prediction of complications within 90 days after surgery

**Fig. 4:**
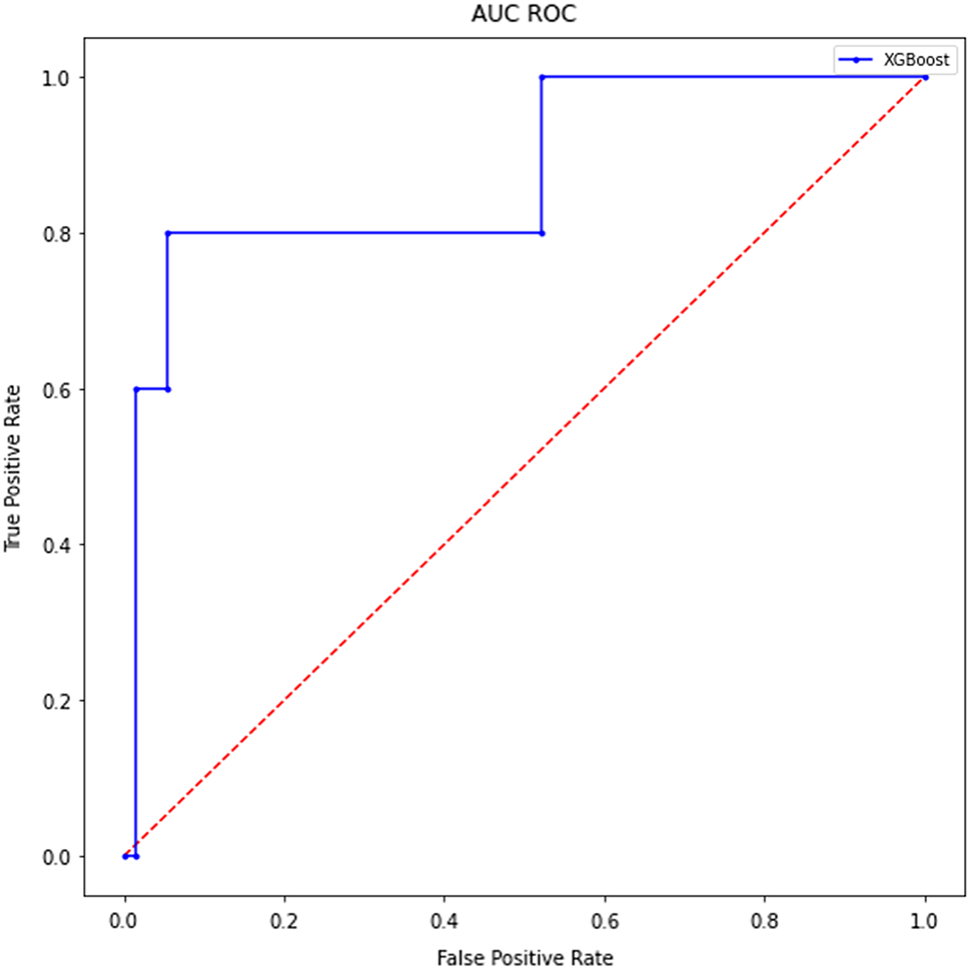
Area under the curve receiver operating characteristics (complications)

**Fig. 5:**
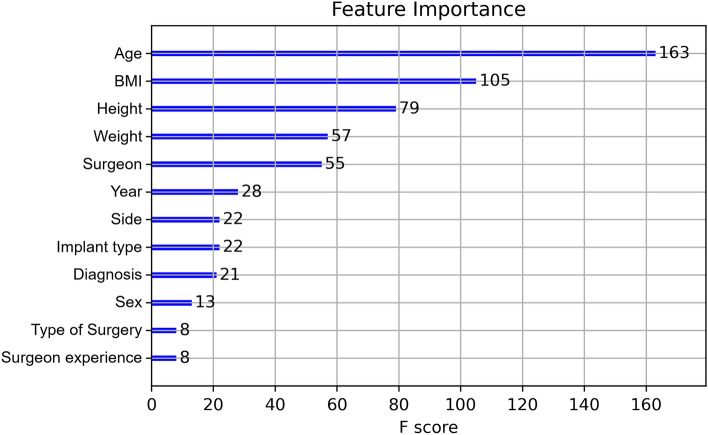
Feature importance of complication prediction

Table 4 demonstrates the accomplished results through various metrics. All results were cross-validated for statistical significance. Additionally, Fig. 4 illustrates the metric AUC in an area under the curve receiver operating characteristics graph. After final computations of the model, a feature importance (Fig. 5) was calculated. The order of parameters according to importance depicts as follows: age, BMI, height, weight, surgeon, year, side, implant type, diagnosis, sex, type of surgery and surgeon experience.

**Table 4:** Results complications

Prediction of complications			[in %]
Accuracy	Sensitivity	Specificity	AUC
92.0	34.8	95.8	78.0

### Prediction of irregular duration of surgery

**Fig. 6:**
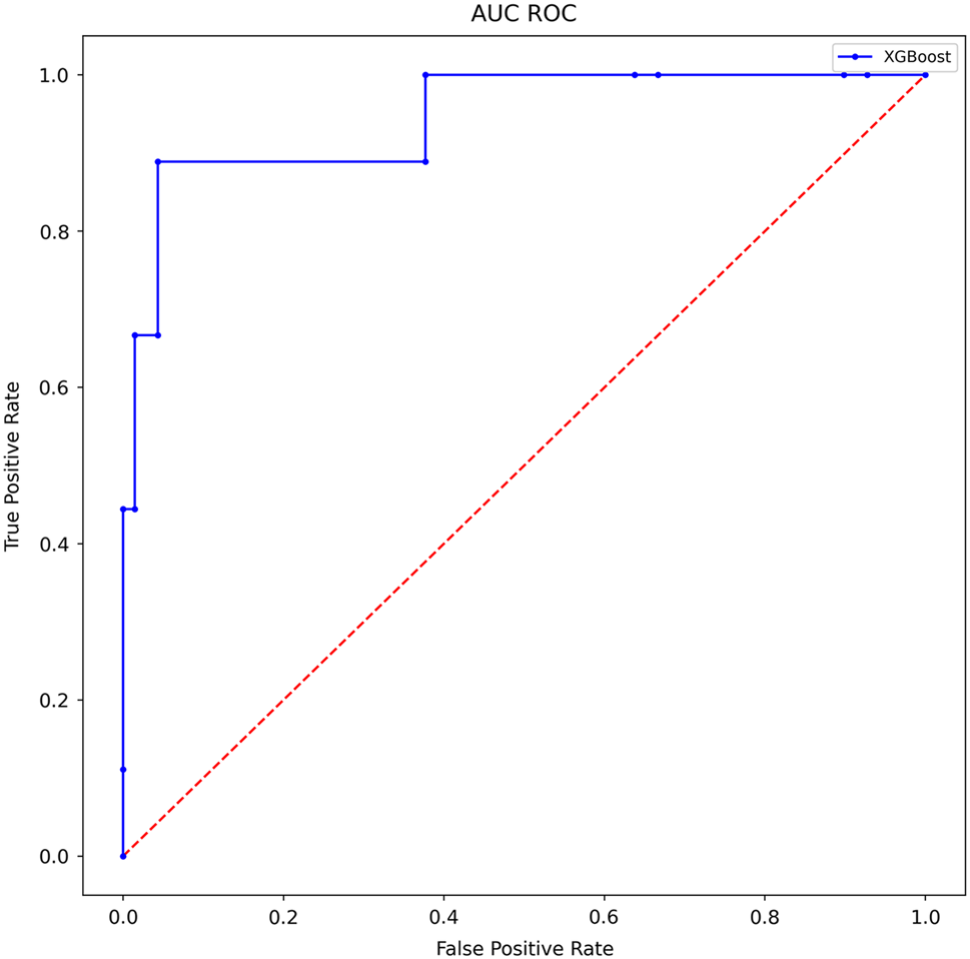
Area under the curve receiver operating characteristics (duration)

**Fig. 7:**
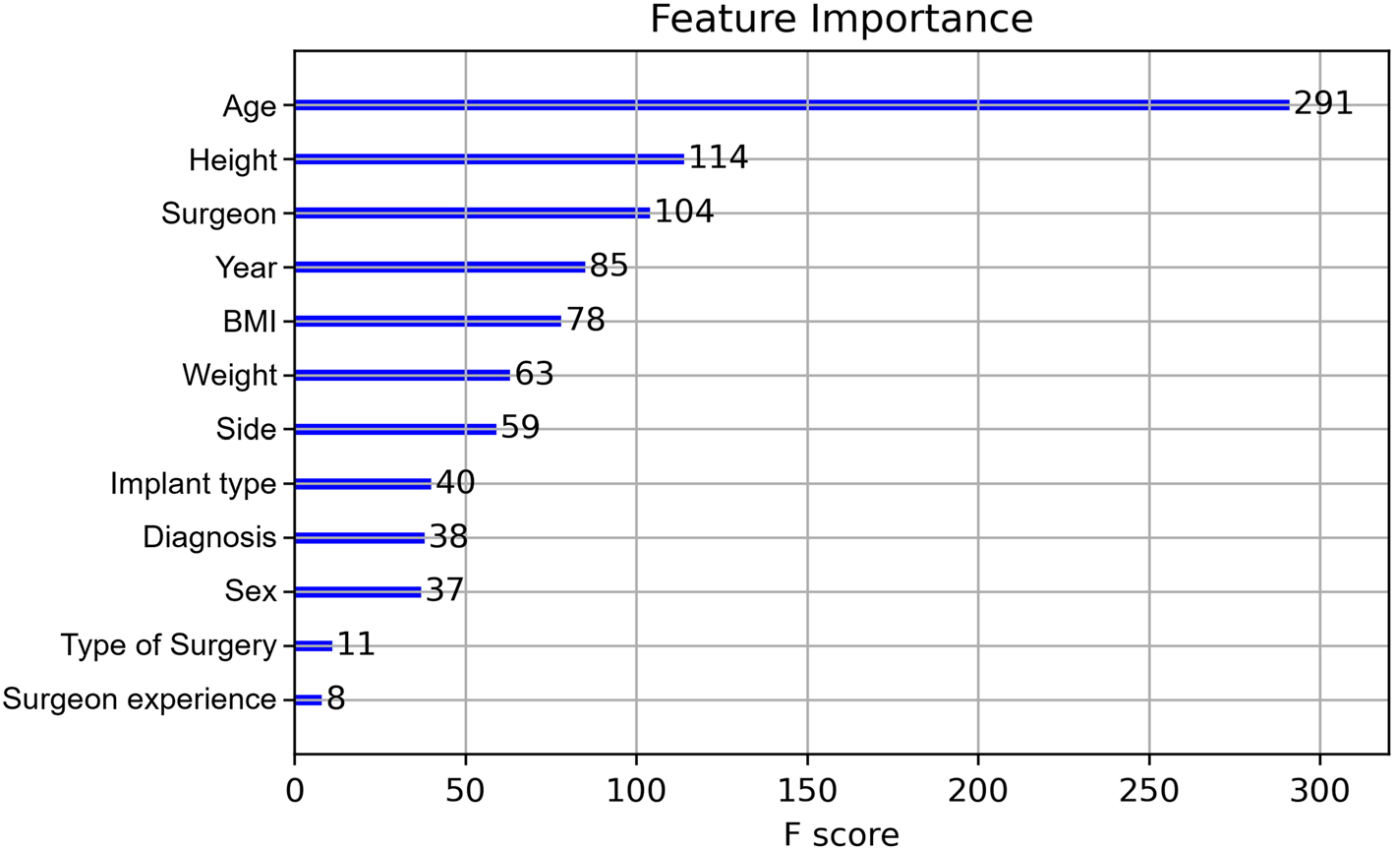
Feature importance of duration prediction

Table 5 demonstrates the accomplished results through various metrics. All results were cross-validated for statistical significance. Additionally, Fig. 6 illustrates the metric AUC in an area under the curve receiver operating characteristics graph. After final computations of the model, a feature importance (Fig. 7) was calculated. The order of parameters according to importance depicts as follows: age, height, surgeon, year, BMI, weight, side, implant type, diagnosis, sex, type of surgery, surgeon experience.

**Table 5:** Results irregular duration

Prediction of irregular duration			[in %]
Accuracy	Sensitivity	Specificity	AUC
93.4	74.0	96.3	91.6

## Discussion

The most important finding of this study is that a highly accurate prediction model was developed with ML and arthroplasty-specific data. The accurate prediction of outcomes in TKA will substantially contribute to personalized treatment algorithms and precision medicine in arthroplasty. Such predictions applying ML models were evaluated in TKA for different variables and yielded heterogeneous results [11, 14, 16, 18]. Harris et al. investigated various ML models (LASSO, gradient boosting, quadratic discriminant analysis) to predict pain and functioning after TKA using PROMs and electronic health record data from three large Veteran Health Administration facilities and achieved fair metrics [14]. Similarly, Li et al. developed a XGBoost model to predict the length of stay after TKA based on 1,826 cases in a single centre and yielded an AUC of 0.74, concluding an improved prediction in comparison to logistic regression models [21]. Recently, Klemt et al. investigated four different ML models on 618 consecutive patients who underwent revision TKA for periprosthetic joint infection to predict recurrent infection and achieved excellent performance across discrimination (AUC range 0.81–0.84) [17]. Kunze et al. examined in a multicentre approach 430 patients and evaluated a Five supervised machine learning algorithms to identify factors for predicting dissatisfaction after primary TKA, yielding a Brier score of 0.082 [19]. On the other side, El Galaly et al. evaluated different ML models (LASSO, random forest, and gradient boosting neural network) to predict early revisions with data from a nationwide arthroplasty registry and concluded that with an AUC of 0.56 to 0.6, none of the models reached the threshold for clinical utility [11]. Furthermore, Pua et al. investigated three ML models (random forest, extreme gradient boosting, and SuperLearner) for predicting walking limitations in a cohort of 4026 patients who underwent primary TKA and concluded moderate results with AUC 0.73 to 0.75 that did not outperform logistic regression [24]. While all studies report similar approaches to examine ML models, their results differ significantly. It was hypothesized that this is due to (1) the limited quality, quantity and complexity of the underlying data and (2) the inappropriate choice of outcome parameters to be predicted. The first two studies obtained their data from health records of few hospitals for very specific objectives, while the latter study applied data from a national registry to investigate failure of TKA in general. ML models are capable to reveal patterns in datasets. However, the dataset must first have sufficient complexity to allow for such distinct patterns. The more complex the investigated pattern or study aim is, the more extensive the input data have to be. A specific data source and architecture built for a ML model with a concise question will therefore likely yield better results than a more extensive data base with a vaguer objective. Hence, the ML model, its outcome labels and the dataset must be attuned to each other. In this context, El Galaly et al. discussed the inclusion of more comprehensive data to increase the accuracy of the ML models to predict revisions of any reason [11]. In this regard, the implementation of data specific to arthroplasty and the exact definition of the objective for the ML model is considered to be crucial to obtain clinical useful results.

Previously, an approach for the application of ML algorithms in TKA was elaborated and using two TKA-specific databases (www.endocert.de, www.eprd.de/en/) was validated in this study. The most important finding of this study is that a feasible ML model to predict outcomes with high accuracy was built. Promising accuracy of 92.0% and 93.4% was achieved for the prediction of complications and irregular surgical duration in this study. Good results with distinct outcome predictions in TKA have already been reported. Katakam et al. aimed to develop ML algorithms for preoperative prediction of prolonged opioid prescriptions after TKA and yielded an AUC of 0.76 [16]. Jo et al. predicted the risk of transfusion using an ML model with an AUC of 0.84 [15]. Similarly, Ko et al. developed a ML model for prediction of postoperative renal failure and yielded an AUC of 0.78 [18]. However, in this approach, first the dataset was evaluated to subsequently conclude which possible outcomes yield useful results in terms of data science methodology and clinical plausibility. While this attunement of input data, ML model and outcome labels allowed for accurate predictions of complications in general and deviations of surgery time in this study, the prediction of distinct complications like thromboembolisms or more general events like revision were not feasible. The overall low data volume was considered a causative factor. Future studies should investigate whether more accurate outcome labels are achievable using this ML approach with increasing data volumes.

The essential importance of an evaluation of various metrics by a data scientist needs to be emphasized. In arthroplasty, it is often the case that the occurrence of an event (e.g. complications in TKA) is very unlikely, but the detection is of crucial importance for the patient. When working with highly imbalanced datasets, algorithms are likely to overfit on the entities with more samples. If the ML algorithm only predicts the high sample entity, the accuracy value might suggest good results, while the algorithm is not capable of predicting the low sample entity cases. Such a considerable class imbalance is present in the dataset at hand with low complication rates in TKA (6.3%). While an accuracy only reflects how many predictions were correct, other metrics give insight into which cases were predicted correctly. In this respect, also lower metric results (sensitivity 34.8% and AUC 78.0% for complications) were obtained. Interestingly, in the case of irregular duration of surgery, the model achieved a very high accuracy (93.4%), specificity (96.3%) and AUC (91.6%). The significant difference compared to complication prediction though is that the sensitivity did not yield values higher than 74.0%. This might partly be explained by the fact, that in this case the dataset was less imbalanced (6.3% vs. 11.5%) and the algorithm was able to better learn both possible outcomes (no irregular duration vs. irregular duration). Hence, the importance to evaluate several elaborate metrics is highlighted when applying ML algorithms in TKA to permit comparability and detailed interpretation. To facilitate a substantive discussion of these mathematical results, collaboration between a data scientist and an orthopaedic surgeon is paramount for the clinical interpretation of the results.

In this study, the feature importance of complication and surgery duration prediction showed that the factors ‘age’, ‘BMI’, ‘height’, ‘weight’ and ‘surgeon’ had the greatest influence on the result of the ML model. An important finding in this study is that these parameters were identified using ML, but have not been confirmed by conventional statistical analysis using a logistic function model. The feature importance indicates to what extent a variable has been weighted in the ML model. However, it does not implicate causality nor unbiased associations. El Galaly et al. described it as a reflection of how the predictions were calculated. It therefore might serve as a reality check of predictive models [11]. Hence, the results yielded by the ML algorithm cannot be directly translated to risk factors per se. While the influence of age and BMI as known risk factors in arthroplasty has already been shown [1, 4, 6, 25], it is interesting to note that the particular surgeon had a major role in this ML model. Noteworthy, the level of experience was examined separately and showed a lower feature importance. If the particular surgeon is a risk factor in reality, is difficult to judge. As already mentioned, the feature importance is not unbiased. It would be possible that in this evaluation, a particular, experienced surgeon was assigned to especially difficult cases with a high failure rate which is subsequently associated to his person. Furthermore, ML is capable of revealing non-linear correlations. In this context, the surgeon may have achieved a high feature importance due to the high occurrence of complications in a specific parameter constellation, i.e. young women with posttraumatic osteoarthritis. However, a severe limitation of ML is the difficulty to interpret the results retrospectively, which is referred to as a “blackbox” problem. The surgeon is weighted as a significant factor in this ML model, but it is hard to retrieve which of the 12 surgeons or which parameter constellations were decisive in this matter. However, these results may be reason for further investigations applying conventional statistical analysis to describe causalities more precisely and to examine whether the discrepancy compared to the results of the logistic function can be explained by, e.g. the small number of cases.

This study has several limitations. Although two arthroplasty-specific registries served as data sources, the data width is still limited as both registries only include a restrictive number of parameters that may affect outcomes. Harris et al. pointed out that the inclusion of more specific data like comorbidity severity or facility complication rates might improve the accuracy of ML models [14]. Both registers are in the process of continuous improvement and are adding new parameters to their evaluations. This analysis was limited to specific parameters that were available for all patients since 2016. However, weight and height were introduced in the registry in 2017. Since these parameters had a relevant Feature Importance despite the missing data points, they were kept in the analysis. While any significant deviations in the missing weight and height parameters are not expected, in principle the results could differ due to the inclusion of these data. Furthermore, EPRD and EndoCert are not designed for evaluation with ML. Therefore, not all parameters were usefully applicable as raw datasets. EPRD provides detailed information of the implants used. If the exact differentiation of manufacturers and particular implant components has a significant impact on the outcome will only become evident with substantially more cases. Therefore, data depth was prioritized instead of data width and generalized this respective information into categorical parameters. This also incorporated meta data into the analysis, which can contribute to a selection bias. The data preparation in this regard required a considerable amount of time. To feed the data into the ML algorithm, the information from EPRD and EndoCert had to be converted and modified, which required an extensive manual screening of the data. This considerable effort is only reasonable in the context of feasibility studies. Prospective, continuous analyses require unique data architecture that is specifically designed for ML. In addition, there are limitations concerning the outcome parameters. Complications are only assessed by EndoCert for 90 days postoperatively and the data are limited to a single hospital. Therefore, some complications may not have been reported.

In this study, the feasibility of the presented ML approach using the available data from two registries specific to arthroplasty was assessed. The future inclusion of currently unavailable specific data for arthroplasty and further cases will most likely improve the accuracy of the ML model and will allow for more specific outcome predictions. Interestingly, Fontana et al. debated that the predictive power across information available at different time points might increase [12, 13]. The input parameters in this study correspond to a baseline at the time of surgery. In this context, Baker et al. investigated patient satisfaction following TKA using a national registry with 22,798 patients and found that postoperative parameters were more predictive than preoperative factors [2]. In this regard, the inclusion of postoperative data might be useful in predicting more complex outcomes such as early revisions. Hence, with more clinically relevant and specific data, the prediction models will become more precise and will allow to determine individual outcome likelihoods, which can be utilized in risk stratifications of treatment algorithms as well as in the informed consent process. Before this can be achieved, however, these ML models must be rigorously tested on larger datasets and in clinical use.

The results of this study show that clinical data can be successfully applied in prediction models using ML. ML may soon become part of clinical practice. To achieve this, pre-and postoperative data should be accumulated in day-by-day clinical work to build a foundation for precise ML algorithms.

## Conclusion

The most important finding of this study is that it was possible to build a feasible ML model to predict outcomes using arthroplasty specific databases. Only high quality and subject-relevant input data will allow drawing valuable conclusions. Therefore, the use of arthroplasty-specific databases is considered to be crucial. Furthermore, the ML model identified relevant parameters, which, however, were not observed in the conventional statistical analysis. An interdisciplinary evaluation and interpretation of these results by a data scientist and an orthopaedic surgeon is paramount to understand the significance of identified parameters and their applicability outside the prediction model.

## References

[1] Arias-de la Torre J, Smith K, Dregan A, Valderas JM, Evans JP, Prieto-Alhambra D, Lozano L, Molina AJ, Martín V, Domingo L, Muñoz L, Espallargues M (2020). Impact of comorbidity on the short- and medium-term risk of revision in total hip and knee arthroplasty. BMC Musculoskelet Disord.

[2] Baker PN, Rushton S, Jameson SS, Reed M, Gregg P, Deehan DJ (2013) Patient satisfaction with total knee replacement cannot be predicted from pre-operative variables alone: A cohort study from the National Joint Registry for England and Wales. Bone Joint J 95-b:1359–1365 10.1302/0301-620X.95B10.32281 24078532

[3] Bentéjac C, Csörgo A, Martínez-Muñoz G (2019) A Comparative Analysis of XGBoost. arXiv:1911.01914

[4] Boyer B, Bordini B, Caputo D, Neri T, Stea S, Toni A (2019). What are the influencing factors on hip and knee arthroplasty survival? Prospective cohort study on 63619 arthroplasties. Orthop Traumatol Surg Res.

[5] Bozic KJ, Kurtz SM, Lau E, Ong K, Chiu V, Vail TP, Rubash HE, Berry DJ (2010). The epidemiology of revision total knee arthroplasty in the United States. Clin Orthop Relat Res.

[6] Charette RS, Sloan M, DeAngelis RD, Lee GC (2019). Higher Rate of Early Revision Following Primary Total Knee Arthroplasty in Patients Under Age 55: A Cautionary Tale. J Arthroplasty.

[7] Chen T, Guestrin C (2016) XGBoost: A Scalable Tree Boosting System. Proceedings of the 22nd ACM SIGKDD International Conference on Knowledge Discovery and Data Mining; San Francisco, California, USA.

[8] Collins GS, Reitsma JB, Altman DG, Moons KGM (2015). Transparent reporting of a multivariable prediction model for individual prognosis or diagnosis (TRIPOD): the TRIPOD Statement. BMC Med.

[9] Delanois RE, Mistry JB, Gwam CU, Mohamed NS, Choksi US, Mont MA (2017). Current epidemiology of revision total knee arthroplasty in the United States. J Arthroplasty.

[10] Edelstein AI, Kwasny MJ, Suleiman LI, Khakhkhar RH, Moore MA, Beal MD, Manning DW (2015). Can the American College of Surgeons Risk Calculator Predict 30-Day Complications After Knee and Hip Arthroplasty?. J Arthroplasty.

[11] El-Galaly A, Grazal C, Kappel A, Nielsen PT, Jensen SL, Forsberg JA (2020). Can machine-learning algorithms predict early revision TKA in the Danish knee arthroplasty registry?. Clin Orthop Relat Res.

[12] Fontana MA (2020). CORR Insights®: can machine-learning algorithms predict early revision TKA in the Danish knee arthroplasty registry?. Clin Orthop Relat Res.

[13] Fontana MA, Lyman S, Sarker GK, Padgett DE, MacLean CH (2019). Can Machine Learning Algorithms Predict Which Patients Will Achieve Minimally Clinically Important Differences From Total Joint Arthroplasty?. Clin Orthop Relat Res.

[14] Harris AHS, Kuo AC, Bowe TR, Manfredi L, Lalani NF, Giori NJ (2021). Can machine learning methods produce accurate and easy-to-use preoperative prediction models of one-year improvements in pain and functioning after knee arthroplasty?. J Arthroplasty.

[15] Jo C, Ko S, Shin WC, Han HS, Lee MC, Ko T, Ro DH (2020). Transfusion after total knee arthroplasty can be predicted using the machine learning algorithm. Knee Surg Sports Traumatol Arthrosc.

[16] Katakam A, Karhade AV, Schwab JH, Chen AF, Bedair HS (2020). Development and validation of machine learning algorithms for postoperative opioid prescriptions after TKA. J Orthop.

[17] Klemt C, Laurencin S, Uzosike AC, Burns JC, Costales TG, Yeo I, Habibi Y, Kwon Y-M (2021) Machine learning models accurately predict recurrent infection following revision total knee arthroplasty for periprosthetic joint infection. Knee Surg Sports Traumatol Arthrosc; 10.1007/s00167-021-06794-3 10.1007/s00167-021-06794-3 34761306

[18] Ko S, Jo C, Chang CB, Lee YS, Moon YW, Youm JW, Han HS, Lee MC, Lee H, Ro DH (2020) A web-based machine-learning algorithm predicting postoperative acute kidney injury after total knee arthroplasty. Knee Surg Sports Traumatol Arthrosc. 10.1007/s00167-020-06258-0 10.1007/s00167-020-06258-0 32880677

[19] Kunze KN, Polce EM, Sadauskas AJ, Levine BR (2020). Development of machine learning algorithms to predict patient dissatisfaction after primary total knee arthroplasty. J Arthroplasty.

[20] Kurtz SM, Ong KL, Lau E, Bozic KJ (2014). Impact of the economic downturn on total joint replacement demand in the United States: updated projections to 2021. J Bone Joint Surg Am.

[21] Li H, Jiao J, Zhang S, Tang H, Qu X, Yue B (2020) Construction and Comparison of Predictive Models for Length of Stay after Total Knee Arthroplasty: Regression Model and Machine Learning Analysis Based on 1826 Cases in a Single Singapore Center. J Knee Surg. 10.1055/s-0040-1710573 10.1055/s-0040-1710573 32512596

[22] Manning DW, Edelstein AI, Alvi HM (2016). Risk prediction tools for hip and knee arthroplasty. J Am Acad Orthop Surg.

[23] Pitta M, Esposito CI, Li Z, Lee Y-y, Wright TM, Padgett DE (2018). Failure after modern total knee arthroplasty: a prospective study of 18,065 knees. J Arthroplasty.

[24] Pua YH, Kang H, Thumboo J, Clark RA, Chew ES, Poon CL, Chong HC, Yeo SJ (2020). Machine learning methods are comparable to logistic regression techniques in predicting severe walking limitation following total knee arthroplasty. Knee Surg Sports Traumatol Arthrosc.

[25] Rassir R, Sierevelt IN, van Steenbergen LN, Nolte PA (2020). Is obesity associated with short-term revision after total knee arthroplasty? An analysis of 121,819 primary procedures from the Dutch Arthroplasty Register. Knee.

[26] Schwartz AM, Farley KX, Guild GN, Bradbury TL (2020). Projections and Epidemiology of Revision Hip and Knee Arthroplasty in the United States to 2030. J Arthroplasty.

[27] Sloan M, Premkumar A, Sheth NP (2018). Projected volume of primary total joint arthroplasty in the U.S., 2014 to 2030. J Bone Joint Surg Am.

[28] Vandenbroucke JP, von Elm E, Altman DG, Gøtzsche PC, Mulrow CD, Pocock SJ, Poole C, Schlesselman JJ, Egger M (2007). Strengthening the reporting of observational studies in epidemiology (STROBE): explanation and elaboration. Epidemiol.

